# Responses of the central nervous system to high linear energy transfer radiation: NSCOR project highlights

**DOI:** 10.1093/jrr/rrt214

**Published:** 2014-03

**Authors:** Gregory Nelson, John Fike, Charles Limoli, André Obenaus, Jacob Raber, Ivan Soltesz, Roman Vlkolinský

**Affiliations:** 1 Loma Linda University; 2 University of California San Francisco; 3 University of California Irvine; 4 Oregon Health and Sciences University

**Keywords:** Brain, High LET radiation, Electrophysiology, Behavior, Neurogenesis

## Abstract

**Overview:** The five-university NSCOR project investigates the responses of the central nervous system to space-like charged particle exposure by evaluating: synaptic function, *in vitro* and *in vivo* neurogenesis, behavior and behaviorally induced gene expression, and oxidative stress of the mouse hippocampus and cultured neural precursor cells. To test the role of reactive oxygen species in mediating the effects of radiation exposure, we compare responses in a catalase overexpressing transgenic mouse strain to wild type. We also use computational models of the hippocampus in three dimensions, informed by experimental measurements, to provide insight into network behavior.

Radiation exposure protocols include single, acute whole-body exposures to ^1^H, ^28^Si and ^56^Fe ions and mixed field exposures using ^1^H + ^56^Fe ions (24 h later). The animal models are 10-week-old C57BL/6J and MCATtg males which are evaluated at 30 and 90 days postirradiation. *In vitro* models are cultured murine and human neural stem cells irradiated with ^1^H, ^16^O, ^28^Si and ^56^Fe ions at multiple energies and are evaluated at times from days to weeks.

**Highlights:** Neural stem cells organized into neurospheres were irradiated with several ions at doses as low as 0.75 cGy. Data show that significant oxidative stress occurs that alters survival, proliferation and differentiation. Overall trends indicate that changes in oxidative stress (persisting for weeks) correlate with particle linear energy transfer (LET). ^56^Fe ions elicited the largest and most persistent changes in stress markers, including antioxidant enzyme expression levels.

The hippocampus-dependent contextual fear conditioning (CFC) and novel object recognition (NOR) paradigms were used to assess cognition and showed cognitive deficits after irradiation with the NOR paradigm more sensitive than CFC. Analysis of neurogenesis indicates that overall neurogenesis is inhibited at doses ≥1 Gy, but newly born activated microglia are significantly elevated at ≥0.1 Gy. High LET radiation affects all lineages of neural precursor cells and elicits a U-shaped dose–response for cells exhibiting the astrocyte marker GFAP. In a mixed field irradiation regimen (0.1 Gy ^1^H, then 0.5 Gy ^56^Fe 24 h later), NOR was impaired with 0.1 Gy ^1^H or 0.1 Gy ^1^H + 0.5 Gy ^56^Fe but not with 0.5 Gy ^56^Fe alone. A negative correlation between newly born activated microglia and NOR or behaviorally activated Arc gene expression was observed for exposures using protons and iron ions, suggesting that neuroinflammation contributes to the cognitive injury. A set of monocyte chemoattractant chemokines was reduced after the mixed beam exposure but not after the individual exposures suggesting compensatory or adaptive responses are elicited by the proton exposure.

Patch clamp recordings on principal neurons of the CA1 and DG hippocampus fields were conducted on mice irradiated with ^1^H, ^28^Si and ^56^Fe iron ions. Input resistance and resting membrane potential were modified by irradiation in CA1 and protons were found to be the most effective ion species. These parameters suggest that more miniature excitatory post synaptic potentials must be elicited simultaneously to initiate action potentials and therefore the neurons are less responsive post irradiation. Si- and Fe-irradiated animals showed only minor alterations in mEPSCs and mIPSCs. Granule neurons of the DG field showed no differences after ^28^Si irradiation, but with ^56^Fe significant increases in AMPA receptor-mediated mEPSC frequency were observed without affecting amplitude. This focuses attention on presynaptic glutamate release mechanisms.

Functional changes in the CA1 network triggered by whole-body irradiation with protons, iron and silicon radiation were assessed with microelectrode array field recordings. Deficits in input–output curves and long-term potentiation (LTP) are observed in proton irradiated mice. In the dentate gyrus field, radiation enhanced input–output curves and LTP which is opposite of the inhibition observed for the CA1 field. This suggests that in the DG the most sensitive targets may be GABA-ergic inhibitory neurons that regulate granular cell excitability. ^28^Si ion effects appear to be associated with dendro-somatic coupling expected to affect signaling of the hippocampal neurons to other brain structures and vary between rostral and ventral hippocampal regions. Observations on MCATtg mice show attenuation of radiation-elicited responses, which implicates reactive oxygen species as mediators of the biological responses.

Modeling activities using a high-fidelity three-dimensional model of the hippocampus have begun and allow simulation of network activities incorporating neuron structural and functional parameters measured experimentally to probe their individual and combined contributions to network behavior. Changes in firing statistics are observed after incorporating measured electrophysiological parameters into the model.

Clinical trial registration number: not applicable.

